# Strength deterioration prediction of pervious concrete in sulfate and dry-wet cycle environments utilizing ultrasonic velocity

**DOI:** 10.1371/journal.pone.0286948

**Published:** 2023-06-13

**Authors:** Hui Song, Shengjie Fan, Shuiliang Zhang, Minghui Gong

**Affiliations:** Jiangxi Provincial Key Laboratory of Hydraulic & Civil Engineering Infrastructure Security, Nanchang Institute of Technology, Nan Chang, Jiangxi Province, China; Middle East Technical University, TURKEY

## Abstract

Strength is a crucial performance indicator for evaluating the durability of pervious concrete (PC). However, there are few models for estimating the remaining strength of in-service PC in sulfate and dry-wet cycle circumstances. Even though there are already direct detection methods for strength, nondestructive testing methods are still worth additional research. This paper aims to give a calculation model for the residual strength of PC under corrosion conditions based on ultrasonic methods, which is economical and convenient for engineering applications. The apparent morphological, compressive strength, and ultrasonic velocity of PC against sulfate and dry-wet cycle attack were examined. The results highlight that the primary cause of the macroscopic mechanical deterioration is the worsening in interface strength. Furthermore, the compressive strength and ultrasonic wave velocity of PC followed the same trends during sulfate and dry-wet cycles, increasing first and subsequently decreasing. Additionally, using the curve-fitting approach, an empirical model of strength deterioration based on ultrasonic velocity was developed and validated utilizing experimental data, demonstrating that the proposed model could more accurately define the strength progression. The results can provide an effective calculation method for monitoring the residual strength of PC pavement engineering in a corrosive environment.

## Introduction

Pervious concrete (PC) is a unique generation of ecologically friendly permeable cement-based material that is gaining popularity. The discontinuity of the aggregate gradation contributes to the larger pore structure of PC compared to traditional ordinary concrete, making this unique pavement material a high permeability, which can effectively improve surface runoff and positively contribute to the prevention and control of urban flooding. Moreover, the interconnected pore structure can effectively reduce the urban ‘heat island effect’, which makes permeable pavement widely used in sponge city construction due to its eco-friendly applicability. [1–3].

There are large amounts of sulfate ions in rainwater and sewage in areas with acid rain disasters. The PC in those areas is likely to be subject to sulfate attack. Acid and sulfate corrosion has occurred on pavements [4–6]. In those areas, coarse aggregate looseness occurs in PC pavement [7, 8]. Spalling of aggregates weakens macro strength, reducing the durability of PC pavement. Therefore, accurate prediction of the remaining concrete strength has a positive significance in estimating the service life of PC pavements.

The micro-pore structure of cement paste has a considerable influence on the overall macroscopic characteristics and corrosion resistance. Adding a certain proportion of additional cementitious ingredients like silica fume, fly ash, and mineral powder to the cement powder can optimize the microscopic pore structure of cement paste [1, 9–12]. Therefore, the penetration of harmful ions can be reduced, thus achieving a valid improvement in the corrosion resistance of traditional ordinary concrete. Similar results can be found in PC, where Bilal et al. [13] showed that silica fume and kaolin could improve the strength of PC and effectively reduce calcium ion precipitation in PC, improving freeze-thaw durability. Zou et al. [14] enhanced the performance of the interfacial transition zone by treating recycled aggregates with silane emulsions to increase freeze-thaw endurance without compromising the permeability of the recycled aggregate pervious concrete. Upgrading the pore structure in cement paste can strengthen PC durability, but most of the previous efforts focused on freeze-thaw resistance. Therefore, further efforts are still needed to evaluate the remaining service life of in-service PC pavement projects under sulfate and dry-wet cycles attack.

However, less emphasis has been made on PC performance developments in sulfate and dry-wet cycle circumstances. Extensive research has been conducted on traditional concrete to ameliorate the protection against corrosion of the identical situations [15–19]. The prediction of PC corrosion resistance learns from methods fortraditional concrete. The deterioration calculation process is mainly based on the chemical product volume or crystalization pressure theory [20–22]. In establishing calculation models, the accelerated diffusion process of harmful ions is characterized by introducing corrosion damage, which is directly related to the formation of chemical reaction products. Yet, the permeability of PC is much higher than ordinary concrete [23]. The distinction in permeability culminates in a distinct difference loss of such two types of concrete strength. Thus, a computational model is necessary to characterize the degradation of PC. Song et al. [24] suggested a chemical-mechanical model to calculate the deterioration subjected to sulfate attack attack based on the damage evolution. However, the parameters in calculation model relied on experimental results, limiting the applicability to different material mixture ratios. Moreover, the strength measurement experiment is a semi-destructive test, which is quite inconvenient for real-time monitoring of the compressive strength of PC projects in service. Ultrasonic nondestructive testing is a common technique for evaluating the performance of pervious concrete [25–27]. Ridengaoqier et al. [28] proposed that the relationship between pervious concrete porosity and ultrasonic wave velocity satisfies a quadratic function. The deterioration of pervious concrete in a corrosive environment causes increased porosity and, as a result, a decrease in strength in the macroscopic mechanical characteristics. However, there is no association between the pervious concrete strength and the ultrasonic wave velocity under a corrosion environment. Thus, monitoring the degree of deterioration of pervious concrete would be substantially simplified by creating a quantitative relationship between these two items.

This paper investigated the compressive strength development and the changes in the appearance and morphology of PC utilizing experimental methods. Meanwhile, ultrasonic wave velocity was examined by employing non-destructive ultrasonic testing. Finally, a predictive computational model relating ultrasonic speed with compressive strength was developed depending on the curve-fitting approach to predict the PC strength deterioration under sulfate and wet-dry cycles attack.

## Materials and methods

### Materials

**Fig 1:**
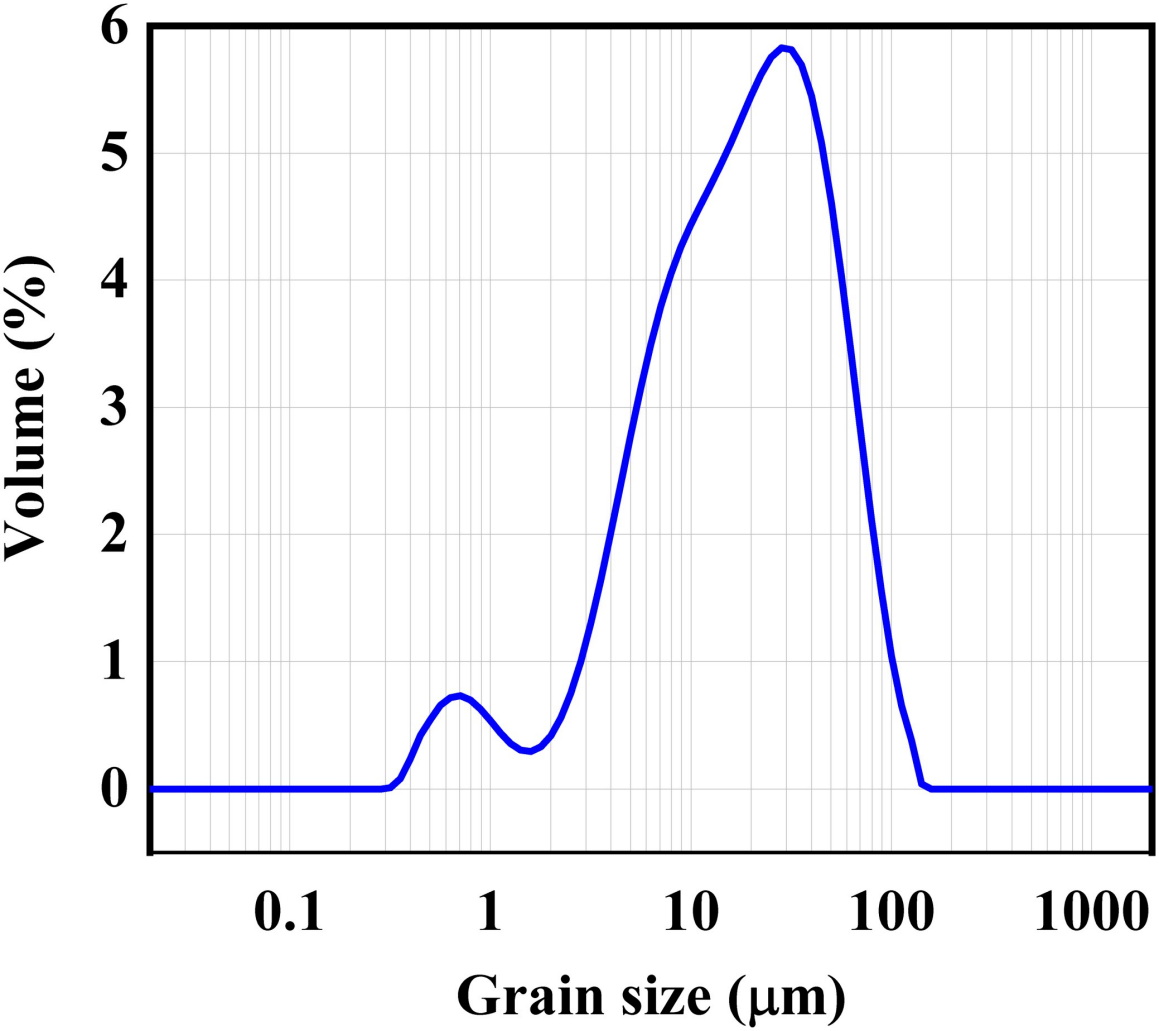
The grain size of cement.

The cementitious material adopted was P.O.45 Portland cement, and the main chemical compositions were examined using X-ray fluorescence, as depicted in Table 1. Fig 1 illustrates the grain particle size of cement with a laser particle size analyzer.

**Table 1:** Composition of cement.

Na_2_O	MgO	Al_2_O_3_	K_2_O	SiO_2_	CaO	Fe_2_O_3_	SO_3_	TiO_2_	MnO
0.548	2.012	8.329	0.790	24.55	53.316	5.821	3.175	0.486	0.323

Single-grade gravel was used as coarse aggregate, aggregate size range is 4.75mm 9.5 mm. The water used was laboratory tap water. A enhancer consisting of nano-silica, water reducer, and retarder, was added to the mixture to replace 4% cement to enhance the strengh of PC with a target porosity of 12%. Table 2 presents the mix ratios.

**Table 2:** Mixture ratio.

Water-cement ratio	Aggregate	Cement	Water	Enhancer
	Kg/m^3^			
0.28	1600	437.2	122.4	17.5
0.31	1600	417.3	129.4	16.7
0.34	1600	399.1	135.7	16.0

**Fig 2:**
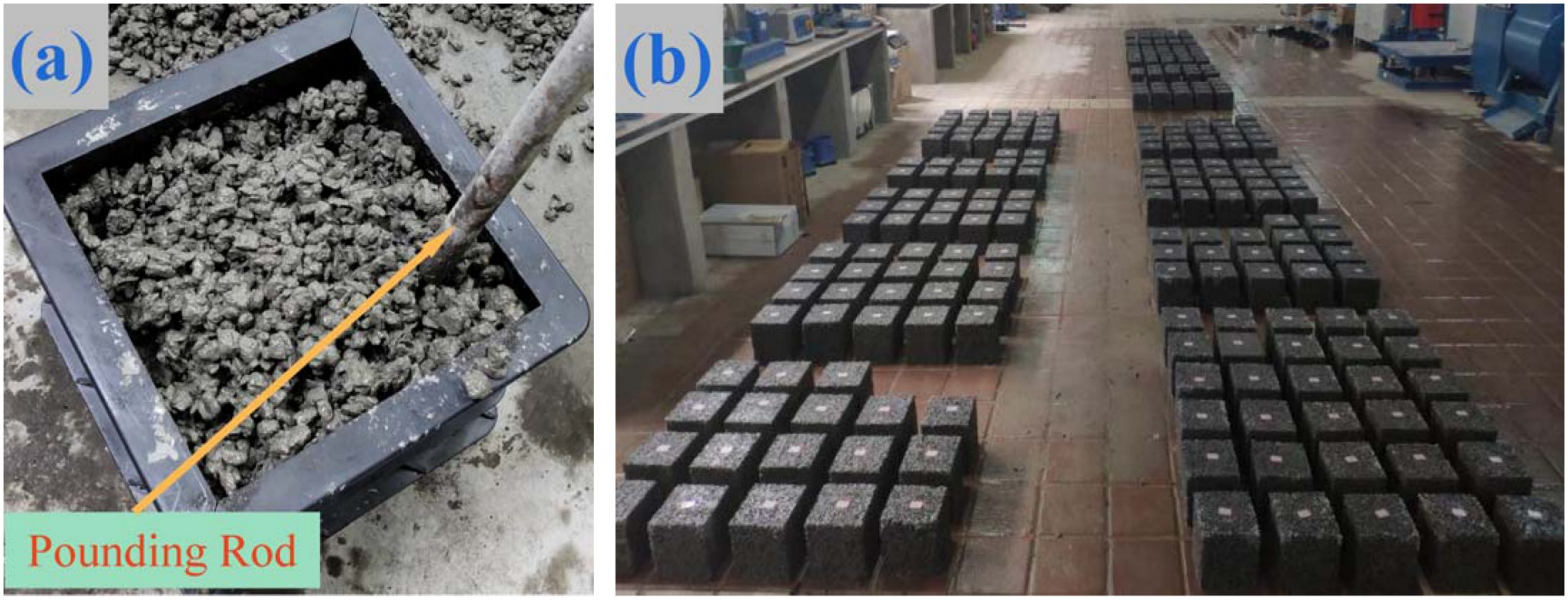
PC samples. (a) Plug molding; (b) Samples after curing.

PC samples are a 150mm × 150mm × 150mm cubic blocks, and the mixture was mixed according to the proportions stated in Table 2 using the cement-coated stone method. Then, the mixture was formed in detachable plastic test molds, and then samples were compacted though insertion molding method. Samples were hammered in three layers, each layer being pounded 20 to 30 times in a spiral pattern from all around to the centre, as illustrated in Fig 2(a). The samples were demoulded after one day of forming. Subsequently, PC samples were positioned inside a concrete-curing chamber set at a curing temperature of 20±2°C and relative humidity of 95%. The tests were conducted following a 28-day curing period according to GB /T 50107–2010 standard [29], and Fig 2(b) illustrates the cured samples.

### Corrosion procedures and experiment methods

**Fig 3:**
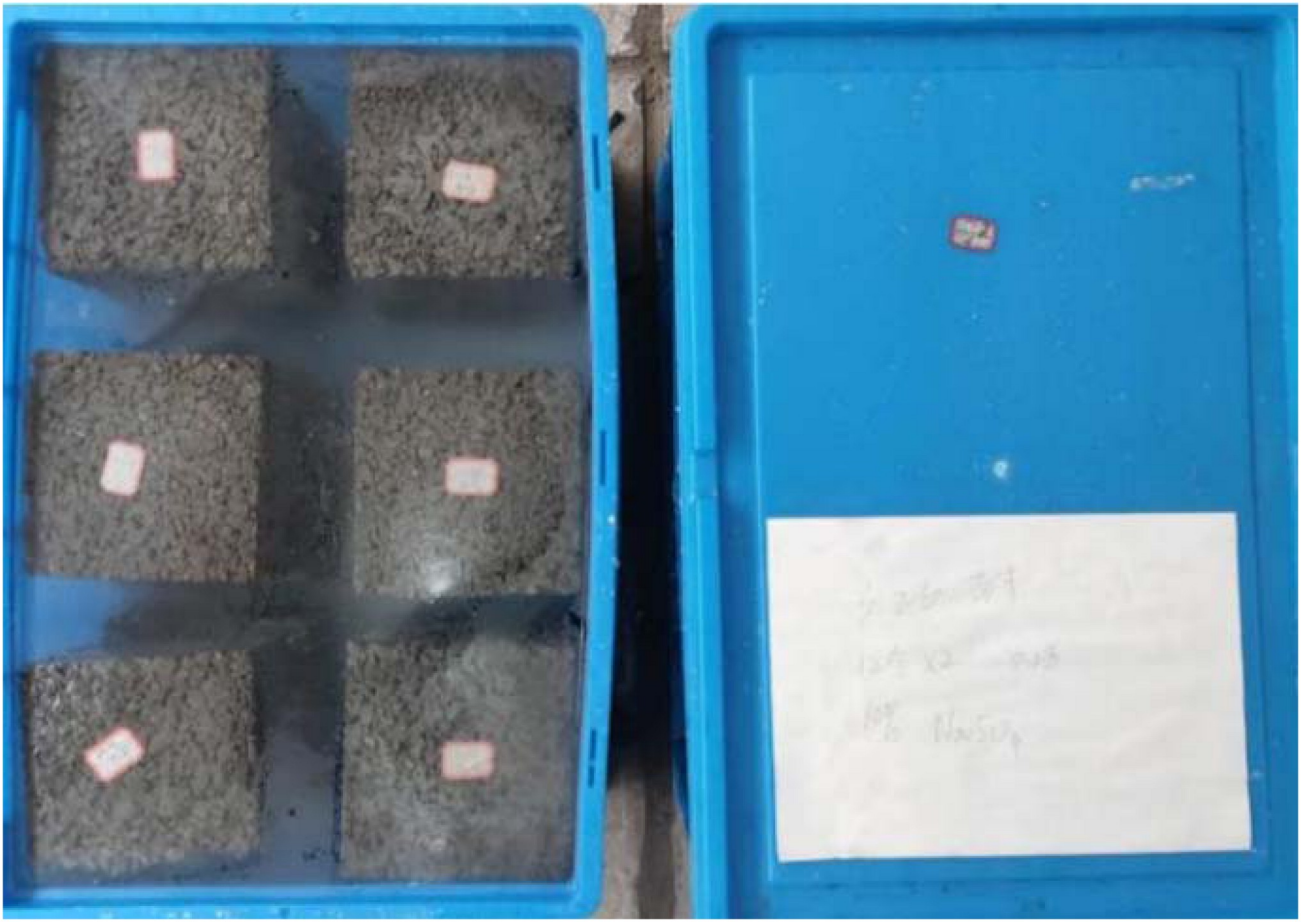
PC samples in the wet state.

The sulfate and wet and dry cycle corrosion process was as follows, firstly, the samples were stored inside a turnover box with a set concentration of sodium sulfate solution, covered with a plastic film, and covered with a lid to prevent water evaporation. Then these samples were immersed in the corrosion solution for five days, as shown in Fig 3. Afterward, the samples were removed and left to dry naturally at room temperature for two days as a cycle of wet and dry cycles. Finally, the PC was tested at the set test time. The mass fraction of the sodium sulphate solution is 3%, 5%, and 8%, respectively, in the wet condition. In order to maintain a comparatively consistent concentration of the corrosion solution, corrosion solution was refreshed every two weeks.

**Fig 4:**
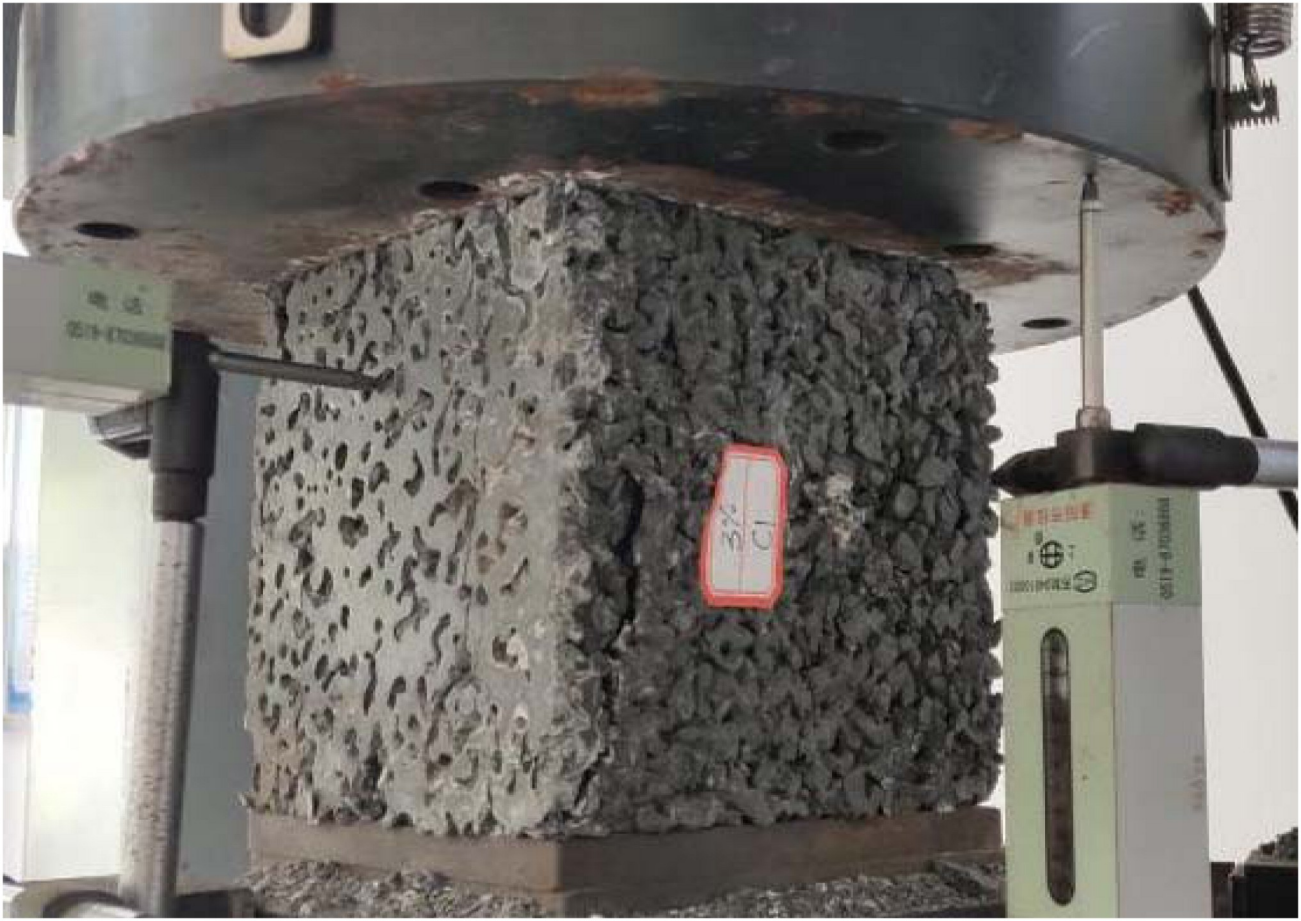
Uniaxial compression test of PC.

Uniaxial compression tests were performed using a microcomputer-controlled hydraulic servo tester according to GB /T 50107–2010 standard [29], as shown in Fig 4. The experiment involves displacement control, and the loading speed is 0.9 mm/min. The compression strength of PC was obtained using peak load *f*_*max*_ (N) by Eq (1),

$$\begin{array}{c} \sigma =\frac{f_{\text{max}}\left(t\right)}{A} \end{array}$$ (1)

A (*mm*^2^) is the surface aera of PC samples.

**Fig 5:**
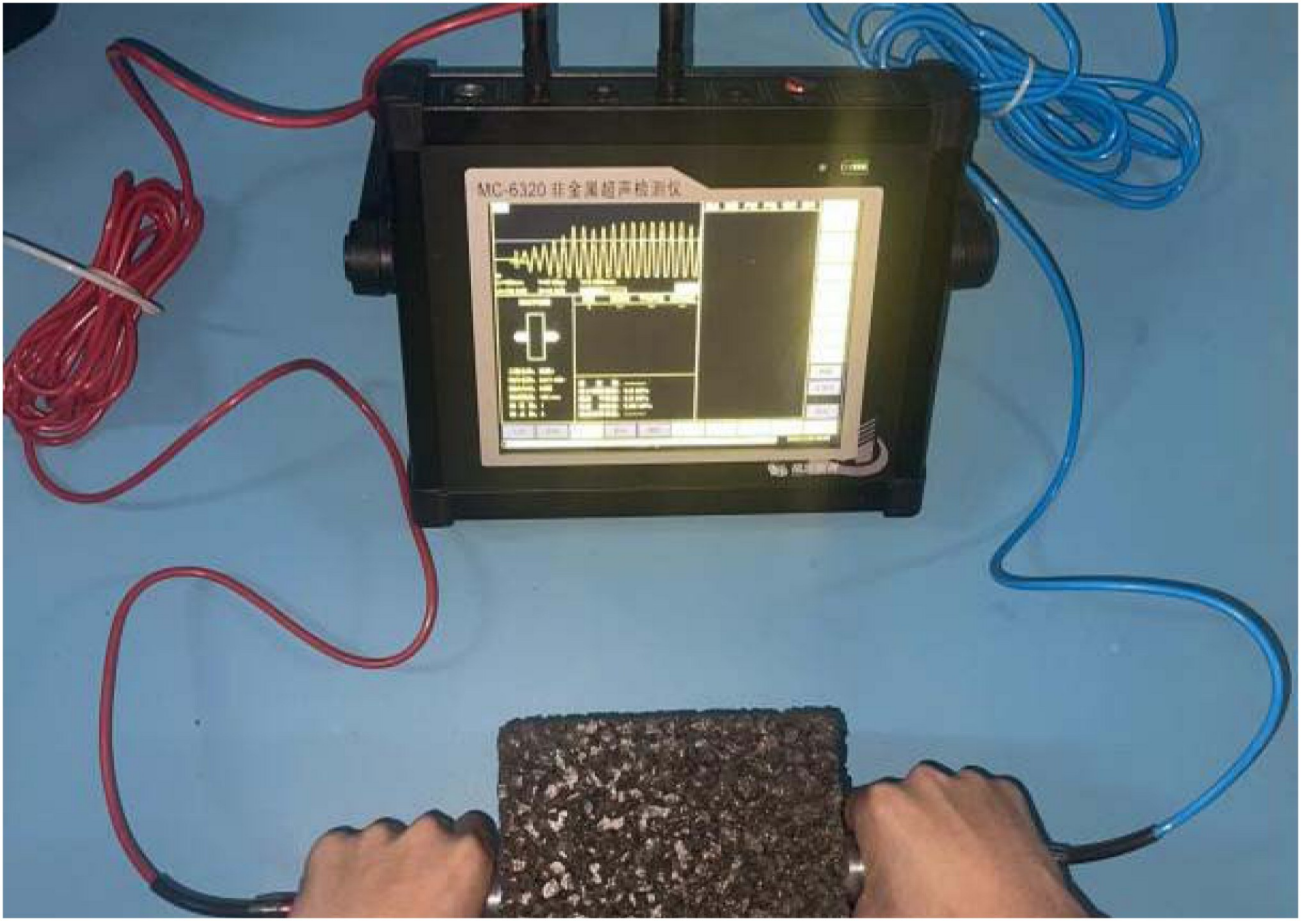
Ultrasonic velocity acquisition by MC-6320 non-metal ultrasonic detector.

Ultrasonic velocity of PC was conducted by a MC-6320 nonmetal ultrasonic detector as shown in Fig 5.

The compression strength and ultrasonic velocity measurements were performed in groups of three samples. The average of the three samples is regarded as the experimental result if the highest or minimum value of compressive strength or ultrasonic velocity does not exceed 20% of the median value. Otherwise, consider the median value to be the outcome of the experiment. In addition, increase the number of samples for the examination again if the gap between the highest and minimum values of the three values and the medium is higher than 20%.

## Experiment results and discussions

### Surface morphology after corrosion

**Fig 6:**
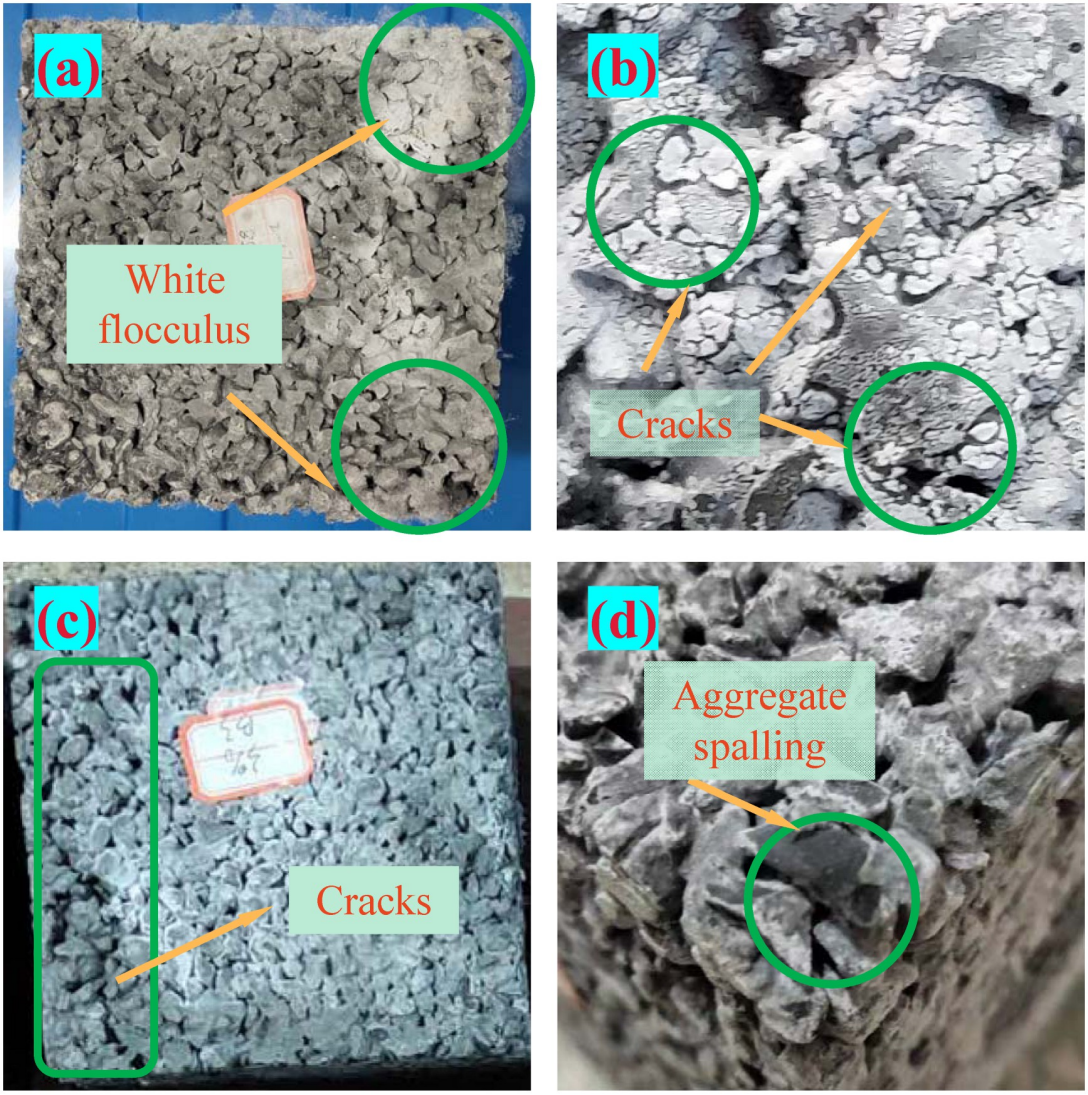
Apparent change of corroded PC.

The deterioration of the PC surface after corrosion is shown in Fig 6.

PC in the dry state, the surface is prone to precipitation of white flocculent material, as shown in Fig 6(a). Concrete subjected to sulfate and dry and wet cycle prone to form expansion chemical products such as delayed ettringite, gypsum, sodium sulfate crystals, and calcium leaching to form calcium carbonate on the surface [19]. The white flocculent was found to be readily soluble in water. However, delayed ettringite, gypsum, and calcium carbonate were not slightly soluble in water. As a result, due to the supersaturated condition that developed in the dry state, the white flocculent is sodium sulphate crystals that precipitate on the surface of PC. The continuous crystallization of sodium sulfate crystals produces crystallization stress upon the pore walls. The crystallization stress further aggravates the damage to pervious concrete [30]. With increasing corrosion time, macroscopic cracks where microcracks converge and connect can be noticed in the cement paste between aggregate interfaces, as presented in Fig 6(b) and 6(c). Spalling of the aggregate occurred during the late stages of erosion, as demonstrated by Fig 6(d). The primary culprit for aggregate spalling is a decline in cement paste bonding qualities brought on by sodium sulfate crystallisation and expansion products. The worsening of the bond strength between the interfaces is a primary cause of the deterioration of the macroscopic mechanical properties [31].

### Compression strength

**Fig 7:**
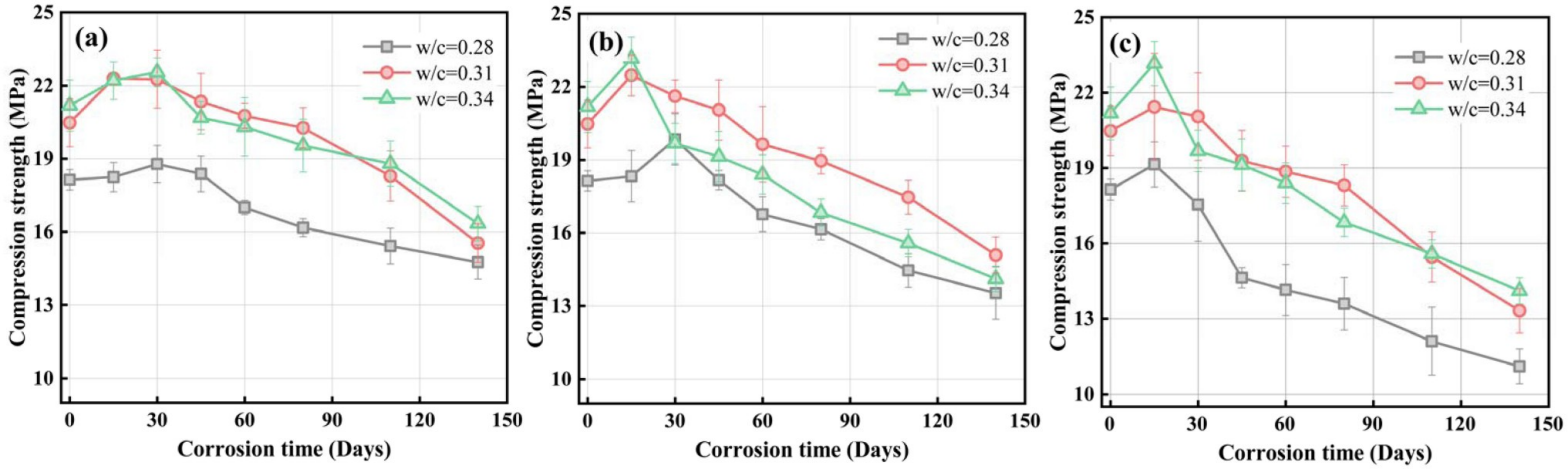
Evolution of compressive strength, (a) 3% Na_2_SO_4_; (b) 5% Na_2_SO_4_; (c) 8% Na_2_SO_4_.

The initial compressive strength of pervious concrete rises with increasing water-cement ratio, as seen in Fig 7. This is distinct from the effect of the water-to-cement mix on conventional concrete. It is generally believed that a lower water-cement ratio makes the transition interface area and the microstructure of the cement paste relatively denser. Therefore, the lower the water-cement ratio in traditional ordinary concrete or high-performance concrete, the higher the strength [32]. However, pervious concrete strength is not only affected by the performance of the microscopic pore structure of the cement paste but also by the intergranular porosity influenced by the rheological properties of the paste [33]. The greater the water-cement ratio, the greater the fluidity of the paste, the lower the intergranular porosity, and the higher the strength of the pervious concrete. Paste rheology has a significant impact on the strength of pervious concrete. The quantitative relationship between paste rheology and intergranular porosity will be the further research work.

PC samples having various water-to-cement ratios exhibit a similar development of strength. According to Fig 7, pervious concrete strength rises first at the initiation of the corrosion stage and then steadily declines until fracture failure. The compressive strength of samples in 3% sodium sulfate solution with a *w/c* ratio of 0.31 increased from 20.47 MPa to 22.29 MPa after 15 days of corrosion, an increase of 8.89%; after 140 days, the compressive strength decreased to 15.36 MPa, a decrease of 24.96% compared to the initial compressive strength. Similar strength variations could be noted on conventional concrete exposed under the same corrosion conditions [11, 15]. However, the strength change rate of PC is significantly higher than conventional portland concrete, mainly due to the enormous differences in the pore structure system between those two types of concrete. The interconnected pores formed between the aggregates can make the permeable concrete have high permeability performance and allow the external sulfate ions quickly invade the interior. The difference in the penetration rate of the erosion ions makes the strength of the PC change faster than that of ordinary concrete. Therefore, PC is more susceptible to corrosion.

Strength deterioration is directly attributed to microscopic pore structure change of cementitious materials [34]. At the initial stages of corrosion, the cement paste reacted with the sulfate ions in a wet state to generate chemical compounds such as ettringite and gypsum, which optimized the initial microscopic pore structure. As a result, the mechanical bonding capabilities of the cement paste were increased, resulting in an improvement in PC strength at the initial stage. As the expansion products accumulated, the capillary pore walls were damaged by the expansion stress of the expansion products. Meanwhile, chemical processes occurred that lowered the concentration of calcium hydroxide and eventually caused calcium breakdown of the CSH gel, which is the major bonding characteristic, causing a decline inside the bonding qualities of aggregate to cement matrix [24]. As a result, the strength of the PC decreased during the later stages of corrosion.

**Fig 8:**
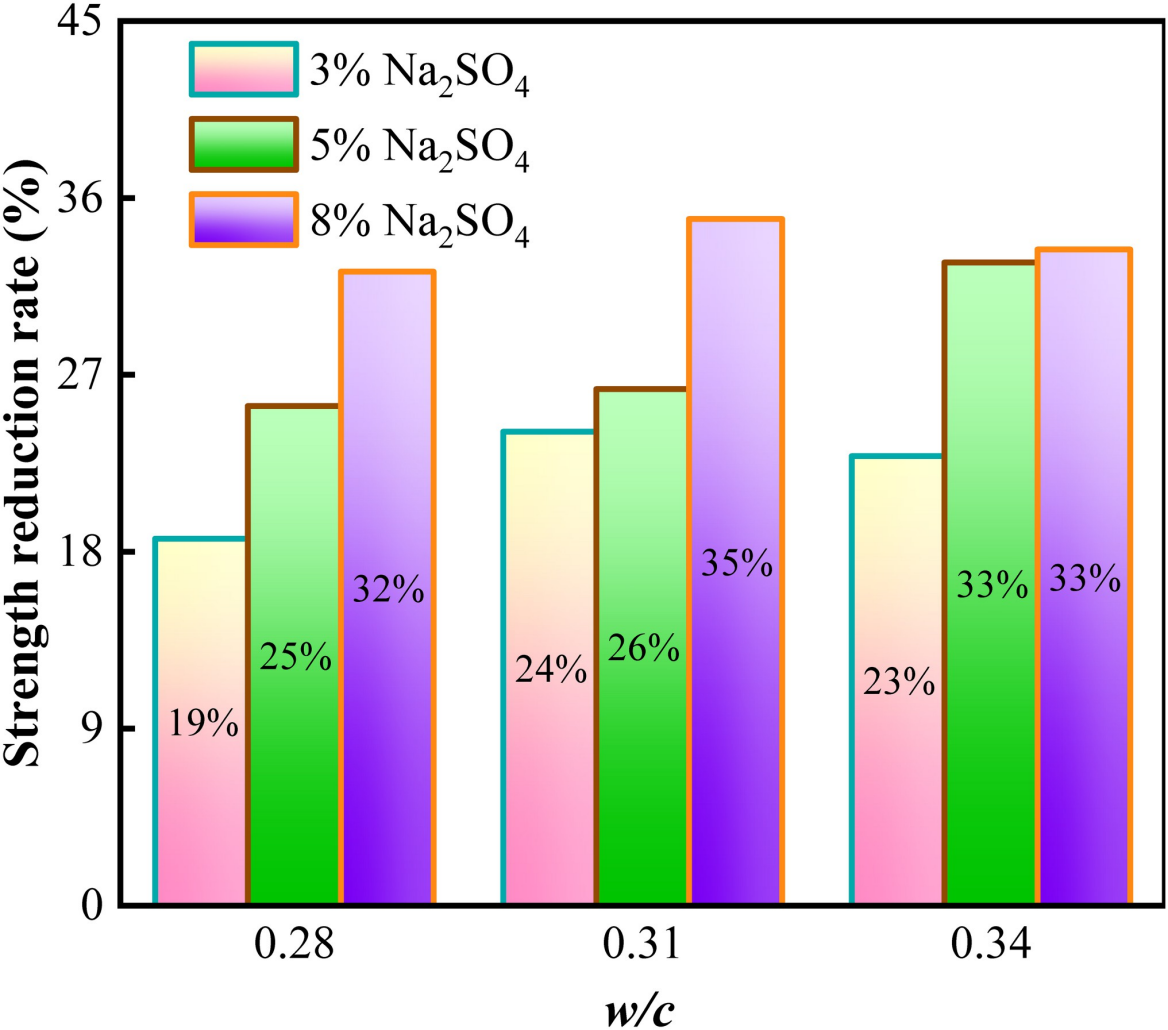
Reduction ratio of compressive strength after 140 days of corrosion.

Fig 8 shows the proportion of PC in different corrosion situations for 140 days compared with the initial strength reduction. The findings revealed that the concentration of corrosion solution or the water-to-cement ratio owns a substantial effect on the strength of PC. Those PC samples with a *w/c* ratio of 0.28 were immersed in 3%, 5%, and 8% sodium sulfate solutions after 140 days, which were 18.65%, 25.4%, and 32.26% lower than the initial strength, respectively. The other two groups of samples with *w/c* ratios 0.31 and 0.34 decreased by 25.4%, 26.28%, 32.71%, and 22.87%, 32.71%, 33.39%, respectively.

The greater concentration of the solution, the greater the concentration gradient formed inside and outside the pore, so sulfate ions seem much more prone to penetrate the pore solution to respond with the hydration product. Therefore, the seriousness of PC strength decline increases with the concentration of the corrosion solution. The greater the water-cement ratio, the greater the decreased compressive strength of pervious concrete in three corrosion solutions. For ordinary concrete, It is commonly recognized that concrete mixture with a low *w/c*ratio has a dense microstructure and is more resistant to sulfate attack [19]. However, the hydration degree of cement paste with a low water-cement ratio is relatively less hydrated than that with a high water-cement ratio. Ongoing hydration on unhydrated cement grains can lessen the detrimental impact of exogenous sulfate ions on the macroscopic strength as the corrosion progresses. The content of unhydrated particles in cement paste of pervious concrete with low water cement ratio is more. The initial microscopic pore structure of pervious concrete with a low water-cement ratio is improved more favourably under the ongoing hydration of unhydrated cement particles. As a result, samples of a larger *w/c* ratio after 140 days, a higher compressive strength loss of PC, and vice versa.

### Ultrasonic velocity

**Fig 9:**
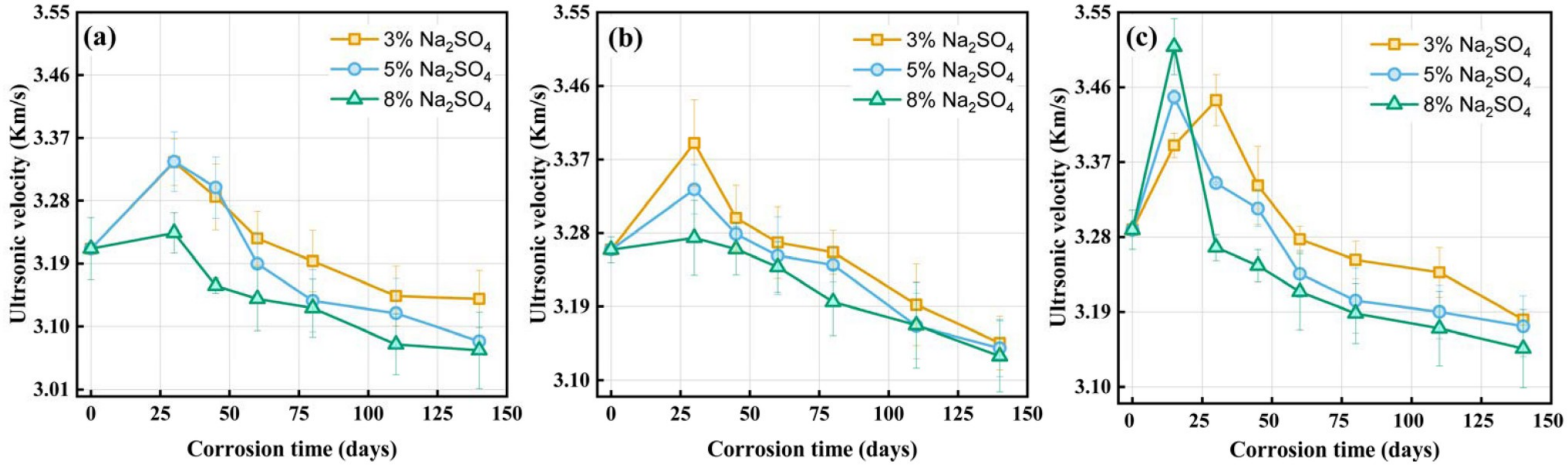
Ultrasonic velocity evolution of PC samples, (a) *w/c*ratio = 0.28; (b) *w/c* ratio = 0.31; (c) *w/c* ratio = 0.34.

Ultrasonic wave is very sensitive to defects and microcracks caused by damage that can be used as an effective non-destructive method for detecting defects caused by chemical corrosion [35]. As illustrated by Fig 9, the ultrasonic wave velocity rises first during the initial corrosion stage. The original defects in pervious concrete were filled with delayed ettringite after a certain period of erosion, making it relatively denser. Therefore, the ultrasonic wave velocity increases. At the later phases of corroding, the dilation product gradually accumulates in capillary pores, the calcium decomposition of the hydration product and the sodium sulfate crystallization pressure cause microcracks, and the bond strength between the aggregates decreases. The ultrasonic wave velocity gets smaller as corrosion time elapses due to the growth in microcrack density. The ultrasonic wave velocity exhibits a similar rise and subsequent drop with corrosion time as the pattern of change in the compressive strength of PC illustrate in Fig 7. Meanwhile, the strength of pervious concrete also rises along with the initial ultrasonic wave velocity as the water-cement ratio increase. The above results show that the ultrasonic wave velocity can reflect the change of compressive strength of pervious concrete.

### Modelling of ultrasonic velocity and compressive strength

Concrete properties alterations are frequently detected utilizing ultrasonic testing methodologies. The propagating path of the ultrasonic wave through the medium would be influenced by the elastic and physical characteristics of the materials [25]. Chandrappa and Biligiri [26] analyzed dynamic elastic modulus Ed though ultrasonic method and proposed a model between *E*_*d*_ and porosity. Compared with the method of obtaining the static elastic modulus by uniaxial compression test, the ultrasonic wave method is a non-destructive test, and the experimental process is simple, which can save time and economic costs. Following is the detail of the connection between *E*_*d*_ and the ultrasonic wave speed *C*(t),

$$\begin{array}{c} E_{d}\left(t\right)=\frac{\left(1+\mu )(1-2\mu \right)}{1-\mu }\rho C{\left(t\right)}^{2} \end{array}$$ (2)

Where *μ* is the Poission’s ratio, taken as 0.25 [26], *ρ* is the density of PC.

Compressive strength is the most basic mechanical feature of concrete. Typically, it is believed that there exists a link among compressive strength with elasticity modulus [36]. The quantitative characterisation association among compressive capacity and the elastic modulus of ordinary or high performance portland cement has been explored and presented empirical models by several specifications and investigations [36–39]. In addition, power functions were often used to quantify the relationship. However, there are few mathematical calculations to chara rize the relationship between these two quantities in PC. We postulate that a power exponential function governs the connection between the strength with static elastic modulus E(t) of PC under corrosion circumstances., i.e.

$$\begin{array}{c} \frac{E(t)}{E_{0}}=\alpha {\left(\frac{f_{c}\left(t\right)}{f_{c0}}\right)}^{\beta } \end{array}$$ (3)

Where *E*_0_ and *f*_*c*0_ are the initial 28 day static modulus and compressive strength, *f*_*c*(*t*)_ is the compressive strength of PC under corrosion condition. *a*, *b* are parameters to be determined.

Since the ultrasonic technique is a non-destructive trying-out methodology, the dynamic elastic modulus acquired using the approach is evaluated in low-stress circumstances. Nonetheless, the static elastic modulus is decided beneath high-stress occasions since it is commonly derived by estimating the slope of the uniaxial compression stress-strain curve at the elasticity segment. In addition, the ultrasonic wavelength is greater than the micro-cracks formed during corrosion, so micro-cracks have little effect on the ultrasonic wave velocity. However, the micro-cracks fashioned through corrosion damage ought to reduce the stiffness of concrete, ensuing in a smaller static elastic modulus. As a result, the static elasticity modulus is typically lower than the dynamic elasticity modulus, as evaluated using ultrasonic methodologies. The association between dynamic and static elastic modulus was frequently defined using linear models [25, 40].

$$\begin{array}{c} E\left(t\right)=\gamma E_{d}\left(t\right) \end{array}$$ (4)

Where *γ* is the proportional coefficient.

Substituting Eq (4) into Eq (3) yields,

$$\begin{array}{c} \frac{E_{d}\left(t\right)}{E_{d0}}=\alpha {\left(\frac{f_{c}\left(t\right)}{f_{c0}}\right)}^{\beta } \end{array}$$ (5)

The connection involving ultrasonic wave velocity and PC strength ought to be determined with the aid of integrating Eq (5) into Eq (2),

$$\begin{array}{c} f_{c}\left(t\right)=f_{c0}{\left(\frac{C{\left(t\right)}^{2}}{\alpha C_{0}^{2}}\right)}^{\frac{1}{\beta }} \end{array}$$ (6)

Where C_0_ is the initial ultrasonic velocity.

**Fig 10:**
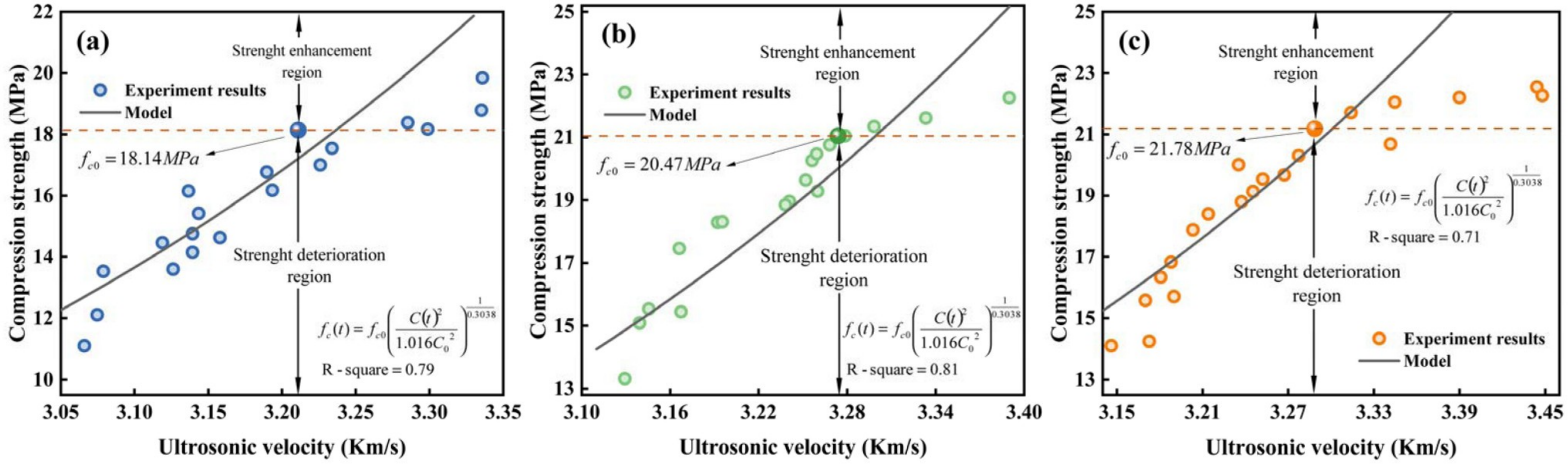
Connection among both PC strength f_*c*_(t) with ultrasonic wave speed *C*(t), (a) *w/c* ratio = 0.28; (b) *w/c* ratio = 0.31; (c) *w/c* ratio = 0.34.

Curvilinear regression analysis of the experiment results of ultrasonic wave velocity and compressive strength using Eq (6), obtaining that *α* is equal to 1.016 and *β* is 0.3038. As illustrated in Fig 10, the interplay among ultrasonic pace and compressive strength of PC for the three *w/c* ratios beneath corrosion circumstances approximately satisfies a power function. The fitted correlation coefficients R-square are 0.79, 0.81, and 0.71, which indicates outcomes from the proposed model correspond satisfactorily with the experiment results.

When the compressive strength exceeds that original value, PC is described as being in the strength-strengthening stage. If not, it has entered a period of strength decline. Fig 10 shows that the predicted compressive strength by ultrasonic wave velocity matches the deterioration stage better than the strength-strengthening stage. According to the theory of crystallization pressure, ettringite first precipitates in pores less than 0.1 um, and fills the pores of CSH gel [41]. Since the filling aperture size is smaller than the ultrasonic wavelength, the ultrasonic wave velocity is not responsive enough to the micropore structure optimization during the early corrosion stage. Further research will focus on the interaction between previous concrete strength and ultrasonic wave during the pore optimization stage. Yet, in general, the model’s calculation value accord well with the strength results in the deterioration stage. Consequently, it is appropriate to forecast how the compressive strength of PC will alter due to the combined effects of sulfate corrosion and wet and dry cycling by paying close attention to the variations in ultrasonic wave speed. To quantify overall mechanical deterioration of PC against sulfate assault, Song et al. [24] constructed a chemical-mechanical deterioration model utilizing chemical kinetic equations and damage mechanics techniques. However, compared with the empirical model proposed in this paper based on the ultrasonic test method, the computational process is relatively more complex and does not facilitate practical engineering applications. The proposed empirical calculation model only requires initial ultrasonic wave velocity and initial compressive strength of PC to be substituted into the calculation. In addition, the prediction results are more accurate.

## Conclusions

The strength deterioration law of PC under the coupled corrosion of dry and wet cycles and sulfate is investigated based on a nondestructive ultrasonic method. The primary goal of the proposed empirical model is to monitor the evolution of PC strength in an aggressive environment and to offer a foundation for estimating the remaining useful lifespan of the PC material. The main findings are as follows,

During sulfate and dry-wet cycling, the bond strength of cement paste between aggregates weakens, resulting in aggregate spalling on the surface of PC. One major factor in the decline of PC compressive strength is the interfacial bond strength of the coarse aggregate.

PC having *w/c* ratios among 0.28 to 0.34 exhibits better residual strength after 140 days of corrosion as the *w/c* ratio rises. Conversely, compared to the original compressive strength, the strength of PC with a lesser *w/c* ratio declined less.

Under corrosion conditions, compressive strength and ultrasonic wave speed show off analogous evolution rules. To illustrate the correlation between PC compressive strength and ultrasonic velocity, a power exponential empirical model was proposed. The experimental findings were applied to evaluate the model and observed that the outcomes of such a model’s computation could more accurately describe the decline of PC compressive strength under corroded circumstances.

## Supporting information

S1 Table Evolution of compressive strength. (DOCX)

S2 Table Ultrasonic velocity evolution of pervious concrete samples. (DOCX)

S3 Table Connection among both pervious concrete strength *f*_*c*(*t*)_ with ultrasonic wave speed. (DOCX)

## References

[1] Wu C, Zhang C, Li J, Wang X, Jiang W, Yang S, et al. A sustainable low-carbon pervious concrete using modified coal gangue aggregates based on ITZ enhancement. J Clean Prod. 2022;377:134310. doi: 10.1016/j.jclepro.2022.134310

[2] He S, Jiao C, Li S. Investigation of mechanical strength and permeability characteristics of pervious concrete mixed with coral aggregate and seawater. Constr Build Mater. 2023;363:129508. doi: 10.1016/j.conbuildmat.2022.129508

[3] Wang H, Li H, Liang X, Zhou H, Xie N, Dai Z. Investigation on the mechanical properties and environmental impacts of pervious concrete containing fly ash based on the cement-aggregate ratio. Constr Build Mater. 2019;202:387–95. doi: 10.1016/j.conbuildmat.2019.01.044

[4] Fu TC, Yeih W, Chang JJ, Huang R. The Influence of Aggregate Size and Binder Material on the Properties of Pervious Concrete. Adv Mater Sci Eng. 2014;2014:1–17. doi: 10.1155/2014/963971

[5] Bashandy AA. Behavior and Durability Evaluation of Recycled Aggregate Pervious Concrete. Acta Technica Napocensis: Civil Engineering & Architecture. 2018;1(61):16–32.

[6] Sahdeo SK, Ransinchung G, Rahul KL, Debbarma S. Reclaimed Asphalt Pavement as a Substitution to Natural Coarse Aggregate for the Production of Sustainable Pervious Concrete Pavement Mixes. J Mater Civil Eng. 2021;33(2):4020469. doi: 10.1061/(ASCE)MT.1943-5533.0003555

[7] Kwiatkowski M. Water quality study of a porous concrete infiltration best management practice: Villanova University; 2004.

[8] Yang B, Gao R D, Xu Q F. Experimental analysis on durability of pervious concrete under complex attack enviroments[J]. Journal of civil, architectural and environmental engineering, 2018, 40(3):53–60. doi: 10.11835/j.issn.1674-4764.2018.03.008

[9] Alyami MH, Mosavi H, Alrashidi RS, Almarshoud MA, Ferraro CC, Riding KA. Lab and Field Study of Physical Sulfate Attack on Concrete Mixtures with Supplementary Cementitious Materials. J Mater Civil Eng. 2021;33(1):4020397. doi: 10.1061/(ASCE)MT.1943-5533.0003500

[10] Faried AS, Mostafa SA, Tayeh BA, Tawfik TA. Mechanical and durability properties of ultra-high performance concrete incorporated with various nano waste materials under different curing conditions. J Build Eng. 2021;43:102569. doi: 10.1016/j.jobe.2021.102569

[11] Yao J, Song H, Yang Y. Correlation Analyses on Physical and Mechanical Parameters of Concrete in Marine Environments. Materials. 2022;15(5):1812. doi: 10.3390/ma15051812 35269042 PMC8912119

[12] Adil G, Kevern JT, Daniel M. Influence of silica fume on mechanical and durability of pervious concrete. Constr Build Mater. 2020;247:118453. doi: 10.1016/j.conbuildmat.2020.118453

[13] Bilal H, Chen T, Ren M, Gao X, Su A. Influence of silica fume, metakaolin & SBR latex on strength and durability performance of pervious concrete. Constr Build Mater. 2021;275:122124. doi: 10.1016/j.conbuildmat.2020.122124

[14] Zou D, Wang Z, Shen M, Liu T, Zhou A. Improvement in freeze-thaw durability of recycled aggregate permeable concrete with silane modification. Constr Build Mater. 2021;268:121097. doi: 10.1016/j.conbuildmat.2020.121097

[15] Yao J, Chen J. Sensitivity analysis of the deterioration of concrete strength in marine environment to multiple corrosive ions. Front Struct Civ Eng. 2022;16(2):175–90. doi: 10.1007/s11709-021-0791-z

[16] Chen J, Qian C, Song H. A new chemo-mechanical model of damage in concrete under sulfate attack. Constr Build Mater. 2016;115:536–43. doi: 10.1016/j.conbuildmat.2016.04.074

[17] Zou DJ, Qin SS, Liu TJ, Jivkov A. Experimental and numerical study of the effects of solution concentration and temperature on concrete under external sulfate attack. Cement Concrete Res. 2021;139:106284. doi: 10.1016/j.cemconres.2020.106284

[18] Zhang ZY, Zhou JT, Yang J, Zou Y, Wang ZS. Understanding of the deterioration characteristic of concrete exposed to external sulfate attack: Insight into mesoscopic pore structures. Constr Build Mater. 2020;260:119932. doi: 10.1016/j.conbuildmat.2020.119932

[19] Chen TW, Chen JK, Chen JH. Weakening-strengthening evolution law of concrete flexural strength under sulfate attack. Int. J. Damage Mech. 2022;31:1187–1211. doi: 10.1177/10567895221095888

[20] Li JP, Xie F, Zhao GW, Li L. Experimental and numerical investigation of cast-in-situ concrete under external sulfate attack and drying-wetting cycles. Constr Build Mater. 2020;249:118789. doi: 10.1016/j.conbuildmat.2020.118789

[21] Zhang X, Qian CX, Chen HC, Liang CY, Kang WC. Calculation of expansion stresses and strains in concrete under sulfate crystallization attack in dry-wet cycles environments. J Mater Civil Eng. 2021;33(3):4020479. doi: 10.1061/(ASCE)MT.1943-5533.0003499

[22] Yin GJ, Zuo XB, Sun XH, Yu TJ. Numerical investigation of the external sulfate attack induced expansion response of cement paste by using crystallization pressure. Model Simul Mater Sc. 2019;27(2):25006. doi: 10.1088/1361-651X/aaf76a

[23] Huang J, Zhang Y, Sun Y, Ren J, Zhao Z, Zhang J. Evaluation of pore size distribution and permeability reduction behavior in pervious concrete. Constr Build Mater. 2021;290:123228. doi: 10.1016/j.conbuildmat.2021.123228

[24] Song H, Yao J, Luo Y, Gui F. A chemical-mechanics model for the mechanics deterioration of pervious concrete subjected to sulfate attack. Constr Build Mater. 2021;312:125383. doi: 10.1016/j.conbuildmat.2021.125383

[25] Ghaffari Moghaddam F, Akbarpour A, Firouzi A. Dynamic modulus of elasticity and compressive strength evaluations of modified reactive powder concrete (MRPC) by non-destructive ultrasonic pulse velocity method. J Asian Archit Build. 2022;21(2):490–9. doi: 10.1080/13467581.2020.1869020

[26] Chandrappa AK, Biligiri KP. Influence of mix parameters on pore properties and modulus of pervious concrete: an application of ultrasonic pulse velocity. Mater Struct. 2016;49(12):5255–71. doi: 10.1617/s11527-016-0858-9

[27] Hoła J, Bień J, Sadowski Ł, Schabowicz K. Non-destructive and semi-destructive diagnostics of concrete structures in assessment of their durability. Bulletin of the Polish Academy of Sciences Technical Sciences. 2015;63(1):87–96. doi: 10.1515/bpasts-2015-0010

[28] Ridengaoqier E, Shigemitsu H, Phommahaxay P, Satoshi K. Experimental study on the porosity evaluation of pervious concrete by using ultrasonic wave testing on surfaces. Constr Build Mater. 2021;300:123959. doi: 10.1016/j.conbuildmat.2021.123959

[29] GB/T 50107—2010, Standard for Evaluation of Concrete Compressive Strength. Beijing: China Architecture & Building Press, 2010.

[30] Wang K, Guo J, Wu H, Yang L. Influence of dry-wet ratio on properties and microstructure of concrete under sulfate attack. Constr Build Mater. 2020;263:120635. doi: 10.1016/j.conbuildmat.2020.120635

[31] Song H, Yao J, Xiang J. The role of aggregate and cement paste in the deterioration of the transitional interface zone of pervious concrete during freeze-thaw cycles. Case Stud Constr Mat. 2022;16:e1086. doi: 10.1016/j.cscm.2022.e01086

[32] Vargas P, Restrepo-Baena O, Tobón JI. Microstructural analysis of interfacial transition zone (ITZ) and its impact on the compressive strength of lightweight concretes. Constr Build Mater. 2017;137:381–9. doi: 10.1016/j.conbuildmat.2017.01.101

[33] Pereira Da Costa FB, Haselbach LM, Da Silva Filho LCP. Pervious concrete for desired porosity: Influence of w/c ratio and a rheology-modifying admixture. Constr Build Mater. 2021;268:121084. doi: 10.1016/j.conbuildmat.2020.121084

[34] Zunino F, Scrivener K. Microstructural developments of limestone calcined clay cement (LC3) pastes after long-term (3 years) hydration. Cement Concrete Res. 2022;153:106693. doi: 10.1016/j.cemconres.2021.106693

[35] Marcantonio V, Monarca D, Colantoni A, Cecchini M. Ultrasonic waves for materials evaluation in fatigue, thermal and corrosion damage: A review. Mech Syst Signal Pr. 2019;120:32–42. doi: 10.1016/j.ymssp.2018.10.012

[36] Yu X, Chen D, Feng J, Zhang Y, Liao Y. Behavior of mortar exposed to different exposure conditions of sulfate attack. Ocean Eng. 2018;157:1–12. doi: 10.1016/j.oceaneng.2018.03.017

[37] Ahmad S, Zubair A, Maslehuddin M. Effect of key mixture parameters on flow and mechanical properties of reactive powder concrete. Constr Build Mater. 2015;99:73–81. doi: 10.1016/j.conbuildmat.2015.09.010

[38] Alsalman A, Dang CN, Prinz GS, Hale WM. Evaluation of modulus of elasticity of ultra-high performance concrete. Constr Build Mater. 2017;153:918–28. doi: 10.1016/j.conbuildmat.2017.07.158

[39] Carrillo J, Ramirez J, Lizarazo-Marriaga J. Modulus of elasticity and Poisson’s ratio of fiber-reinforced concrete in Colombia from ultrasonic pulse velocities. J Build Eng. 2019;23:18–26. doi: 10.1016/j.jobe.2019.01.016

[40] Trifone L. A study of the correlation between static and dynamic modulus of elasticity on different concrete mixes:West Virginia University, Morgantown, West Virginia; 2017.

[41] Zuo XB, Sun W, Yu C. Numerical investigation on expansive volume strain in concrete subjected to sulfate attack. Constr Build Mater. 2012;36:404–10. doi: 10.1016/j.conbuildmat.2012.05.020

