# Supplementary material for: Strength deterioration prediction of pervious concrete in sulfate and dry-wet cycle environments utilizing ultrasonic velocity
Source: PLoS One. 2023 Jun 13;18(6):e0286948. doi: 10.1371/journal.pone.0286948 (PMC10263306; doi:10.1371/journal.pone.0286948)
Supplement: S1 Table — (DOCX) [file pone.0286948.s001.docx]

**Table 1. Evolution of compressive strength**

| **Solution concentration** | **Corrosion times (days)** | **w/c=0.28** | | **w/c=0.31** | | **w/c=0.34** | |
| --- | --- | --- | --- | --- | --- | --- | --- |
|  |  | **Compression strength (MPa)** | **Standard Deviation** | **Compression strength (MPa)** | **Standard Deviation** | **Compression strength (MPa)** | **Standard Deviation** |
| 3% Na_2_SO_4_ | 0 | 18.1367 | 0.42253 | 20.47 | 0.98534 | 21.1775 | 1.05304 |
|  | 15 | 18.2433 | 0.60352 | 22.28667 | 0.24007 | 22.2 | 0.76864 |
|  | 30 | 18.7833 | 0.7635 | 22.25667 | 1.18462 | 22.54333 | 0.58825 |
|  | 45 | 18.3767 | 0.73364 | 21.34333 | 1.15001 | 20.67667 | 0.67099 |
|  | 60 | 17 | 0.28583 | 20.75667 | 0.51316 | 20.30667 | 1.19684 |
|  | 80 | 16.1733 | 0.37018 | 20.26 | 0.82164 | 19.535 | 1.08503 |
|  | 110 | 15.4133 | 0.73105 | 18.28567 | 1.02758 | 18.79687 | 0.92589 |
|  | 140 | 14.7533 | 0.70059 | 15.5356 | 0.79011 | 16.335 | 0.70995 |
| 5% Na_2_SO_4_ | 0 | 18.13667 | 0.42253 | 20.47 | 0.98534 | 21.1775 | 1.05304 |
|  | 15 | 18.32667 | 1.06171 | 22.47 | 0.84077 | 23.15667 | 0.88189 |
|  | 30 | 19.84333 | 1.04902 | 21.61333 | 0.67144 | 19.67333 | 0.82033 |
|  | 45 | 18.16667 | 0.40067 | 21.04333 | 1.23654 | 19.13 | 1.03184 |
|  | 60 | 16.76333 | 0.71557 | 19.63667 | 1.54907 | 18.39667 | 0.80872 |
|  | 80 | 16.14667 | 0.43317 | 18.955 | 0.53201 | 16.83 | 0.55561 |
|  | 110 | 14.45667 | 0.68537 | 17.45894 | 0.69851 | 15.57427 | 0.56361 |
|  | 140 | 13.53 | 1.07615 | 15.09 | 0.74505 | 14.10667 | 0.5232 |
| 5% Na_2_SO_4_ | 0 | 18.13667 | 0.42253 | 20.47 | 0.98534 | 21.1775 | 1.05304 |
|  | 15 | 19.14 | 0.89538 | 21.43 | 2.11773 | 23.15667 | 0.88189 |
|  | 30 | 17.54 | 1.46031 | 21.04667 | 1.7403 | 19.67333 | 0.82033 |
|  | 45 | 14.62667 | 0.40278 | 19.28 | 1.20503 | 19.13 | 1.03184 |
|  | 60 | 14.14333 | 1.0155 | 18.85 | 1.02276 | 18.39667 | 0.80872 |
|  | 80 | 13.59333 | 1.04548 | 18.30667 | 0.8247 | 16.83 | 0.55561 |
|  | 110 | 12.10667 | 1.34675 | 15.44474 | 1.00077 | 15.57427 | 0.56361 |
|  | 140 | 11.10667 | 0.69616 | 13.32 | 0.8858 | 14.10667 | 0.5232 |
