# Supplementary material for: Strength deterioration prediction of pervious concrete in sulfate and dry-wet cycle environments utilizing ultrasonic velocity
Source: PLoS One. 2023 Jun 13;18(6):e0286948. doi: 10.1371/journal.pone.0286948 (PMC10263306; doi:10.1371/journal.pone.0286948)
Supplement: S2 Table — (DOCX) [file pone.0286948.s002.docx]

**Table 2. Ultrasonic velocity evolution of pervious concrete samples**

| **Solution concentration** | **Corrosion times (days)** | **w/c=0.28** | | **w/c=0.31** | | **w/c=0.34** | |
| --- | --- | --- | --- | --- | --- | --- | --- |
|  |  | **Ultrasonic velocity (Km/s)** | **Standard Deviation** | **Ultrasonic velocity (Km/s)** | **Standard Deviation** | **Ultrasonic velocity (Km/s)** | **Standard Deviation** |
| 3% Na_2_SO_4_ | 0 | 3.21111 | 0.04454 | 3.21111 | 0.04454 | 3.21111 | 0.04454 |
|  | 30 | 3.33533 | 0.03347 | 3.336 | 0.04284 | 3.234 | 0.02901 |
|  | 45 | 3.28533 | 0.04736 | 3.29889 | 0.04414 | 3.158 | 0.01102 |
|  | 60 | 3.226 | 0.03894 | 3.18978 | 0.04316 | 3.13933 | 0.04547 |
|  | 80 | 3.19356 | 0.044 | 3.13667 | 0.04452 | 3.12622 | 0.04134 |
|  | 110 | 3.14356 | 0.04286 | 3.11889 | 0.04985 | 3.07467 | 0.04356 |
|  | 140 | 3.13933 | 0.04081 | 3.07889 | 0.01935 | 3.066 | 0.05468 |
| 5% Na_2_SO_4_ | 0 | 3.25933 | 0.01604 | 3.25933 | 0.01604 | 3.25933 | 0.01604 |
|  | 30 | 3.39 | 0.05288 | 3.33333 | 0.03014 | 3.274 | 0.04585 |
|  | 45 | 3.29822 | 0.04025 | 3.27867 | 0.0094 | 3.26 | 0.03143 |
|  | 60 | 3.26822 | 0.04375 | 3.252 | 0.04756 | 3.23822 | 0.03103 |
|  | 80 | 3.25622 | 0.027 | 3.24089 | 0.02102 | 3.19533 | 0.04068 |
|  | 110 | 3.19222 | 0.04993 | 3.16622 | 0.04011 | 3.16733 | 0.05236 |
|  | 140 | 3.14556 | 0.03272 | 3.13933 | 0.03497 | 3.12933 | 0.04347 |
| 8% Na_2_SO_4_ | 0 | 3.28867 | 0.02359 | 3.28867 | 0.02359 | 3.28867 | 0.02359 |
|  | 15 | 3.39 | 0.01467 | 3.448 | 0.00867 | 3.50867 | 0.03376 |
|  | 30 | 3.44422 | 0.03068 | 3.34467 | 0.00115 | 3.26733 | 0.01568 |
|  | 45 | 3.34178 | 0.04702 | 3.314 | 0.02163 | 3.24533 | 0.01954 |
|  | 60 | 3.27733 | 0.01538 | 3.23533 | 0.02835 | 3.214 | 0.04586 |
|  | 80 | 3.25222 | 0.02271 | 3.20333 | 0.03908 | 3.188 | 0.03591 |
|  | 110 | 3.23733 | 0.03003 | 3.19 | 0.03188 | 3.17 | 0.04468 |
|  | 140 | 3.18067 | 0.00677 | 3.17267 | 0.03637 | 3.146 | 0.04682 |
