# Supplementary material for: Strength deterioration prediction of pervious concrete in sulfate and dry-wet cycle environments utilizing ultrasonic velocity
Source: PLoS One. 2023 Jun 13;18(6):e0286948. doi: 10.1371/journal.pone.0286948 (PMC10263306; doi:10.1371/journal.pone.0286948)
Supplement: S3 Table — (DOCX) [file pone.0286948.s003.docx]

**Table 3. Connection among both pervious concrete strength *f*_c_(t) with ultrasonic wave speed**

| **w/c=0.28** | | **w/c=0.31** | | **w/c=0.34** | |
| --- | --- | --- | --- | --- | --- |
| **Ultrasonic velocity (Km/s)** | **Compression strength (MPa)** | **Ultrasonic velocity (Km/s)** | **Compression strength (MPa)** | **Ultrasonic velocity (Km/s)** | **Compression strength (MPa)** |
| 3.21111 | 18.1367 | 3.25933 | 20.47 | 3.28867 | 21.1775 |
| 3.33533 | 18.7833 | 3.39 | 22.25667 | 3.39 | 22.2 |
| 3.28533 | 18.3767 | 3.29822 | 21.34333 | 3.44422 | 22.54333 |
| 3.226 | 17 | 3.26822 | 20.75667 | 3.34178 | 20.67667 |
| 3.19356 | 16.1733 | 3.25622 | 20.26 | 3.27733 | 20.30667 |
| 3.14356 | 15.4133 | 3.19222 | 18.28567 | 3.25222 | 19.535 |
| 3.13933 | 14.7533 | 3.14556 | 15.5356 | 3.23733 | 18.79687 |
| 3.21111 | 18.13667 | 3.25933 | 20.47 | 3.18067 | 16.335 |
| 3.336 | 19.84333 | 3.33333 | 21.61333 | 3.28867 | 21.1775 |
| 3.29889 | 18.16667 | 3.27867 | 21.04333 | 3.448 | 22.26 |
| 3.18978 | 16.76333 | 3.252 | 19.63667 | 3.34467 | 22.04667 |
| 3.13667 | 16.14667 | 3.24089 | 18.955 | 3.314 | 21.70667 |
| 3.11889 | 14.45667 | 3.16622 | 17.45894 | 3.23533 | 20.00667 |
| 3.07889 | 13.53 | 3.13933 | 15.09 | 3.20333 | 17.88 |
| 3.21111 | 18.13667 | 3.25933 | 20.47 | 3.19 | 15.70433 |
| 3.234 | 17.54 | 3.274 | 21.04667 | 3.17267 | 14.25 |
| 3.158 | 14.62667 | 3.26 | 19.28 | 3.28867 | 21.1775 |
| 3.13933 | 14.14333 | 3.23822 | 18.85 | 3.50867 | 23.15667 |
| 3.12622 | 13.59333 | 3.19533 | 18.30667 | 3.26733 | 19.67333 |
| 3.07467 | 12.10667 | 3.16733 | 15.44474 | 3.24533 | 19.13 |
| 3.066 | 11.10667 | 3.12933 | 13.32 | 3.214 | 18.39667 |
|  |  |  |  | 3.188 | 16.83 |
|  |  |  |  | 3.17 | 15.57427 |
|  |  |  |  | 3.146 | 14.10667 |
